# The rollout of the COVID-19 vaccination: what can Canada learn from Israel?

**DOI:** 10.1186/s13584-021-00449-x

**Published:** 2021-02-17

**Authors:** Gregory P. Marchildon

**Affiliations:** Institute of Health Policy, Management and Evaluation, University of Toronto, 155 College Street, Suite 425, Toronto, Ontario M5T 3M6 Canada

**Keywords:** COVID-19, Canada, Israel, Vaccination, Governance, Federalism

## Abstract

This commentary compares Israel’s COVID-10 vaccination response to the much slower and less successful vaccination campaign in Canada. Although Canada did start with some structural disadvantages relative to Israel including less centralized and coherent emergency planning and a more complex demographic geography, there are, nonetheless, some important policy lessons Canada can draw from Israel. These include a more strategic use of national leadership in the vaccination campaign and the greater use of primary care resources and providers.

## Israel’s success and Canada’s failure?

As Bruce Rosen, Ruth Waitzberg, and Avi Israeli make clear, Israel has been, at least to the date of writing this commentary, very successful – indeed a very positive outlier – in rapidly administering the COVID-19 vaccination to its population [1]. As can be seen in Table 1, on Israel vastly outstrips North America (United States, Canada and Mexico) and all of the higher-income countries of Western Europe in terms of the number of COVID-19 vaccination doses administered per 100 people as of 11 February 2021. In contrast, Canada not only has a vaccine rate far well below Israel but also well below the United States and the United Kingdom, countries to which Canada’s system of public health and universal health coverage is most often compared. When compared to other higher-income OECD countries, Canada’s overall performance in vaccinating its population started out mediocre in early January and has been deteriorating since [2].

**Table 1:** Cumulative COVID-19 vaccination doses administered per 100 people by Feb. 1, 2021, or latest date available

*Country*	*Doses*^a^	*Country*	*Doses*^a^
**Israel**	**71.2**	Finland	4.5
United Kingdom	20.7	Belgium	4.4
United States	14.0	Germany	4.4
Denmark	6.3	Sweden	4.3
Iceland	5.2	Portugal	4.3
Spain	5.0	France	3.8
Ireland	4.9	Austria	3.8
Switzerland	4.8	**Canada**	**3.1**
Norway	4.7	Netherlands	2.9
Italy	4.7	Mexico	0.6

Source: Our World in Data coronavirus pandemic data explorer: https://ourworldindata.org/coronavirus

^a^Counted as a single dose as opposed to single vaccination involving multiple doses

The ongoing controversy in Canada concerning vaccine procurement, supply and distribution also reflects the feeling that Canada is not doing as well in its vaccine campaign as it should be [3]. Public health experts have been left wondering if the Government of Canada moved rapidly enough to guarantee a sufficient and timely supply of vaccines. This criticism has been amplified, and at times exaggerated, by provincial governments who have disparaged the federal government for its procurement efforts thus far [4]. In addition, media reports have fuelled the perception of Canada’s vulnerability given the country's dependence on foreign suppliers due to the loss of domestic vaccine production capacity in the last half century [5].

## Israel’s natural advantages and differences relative to Canada

I will first review some of the Israel’s natural advantages that have allowed it to so outperform Canada in terms of the roll out of COVID-19 vaccination. These factors are mentioned by Rosen and colleagues but I will try to put them in rough order of importance in terms of Canada. The first and most obvious is the fact that Canada’s population is dispersed over a vast geography that poses acute challenges in distribution. Although this factor is attenuated by an urban population concentrated near the US border, there remains a large number of Canadians living in rural and remote areas that are distant from these urban centres. When this factor is combined with the highly decentralized nature of the Canadian federation, this results in time lags in distribution.

While the Government of Canada has taken on the responsibility of procuring vaccines, it is the provincial and territorial (PT) governments which actually administer vaccinations to the residents in their respective jurisdictions based on independently determined criteria for priority. This means that the federal government has a central point in each of the 13 provinces and territories which it delivers the vaccines. The PT governments must then distribute the vaccine to countless points within their own geographies thereby creating a chronic gap between the doses delivered by the federal government and the doses actually administered to Canadians by PT governments: as of 25 January 2021, this meant that slightly less than 75% of the doses delivered to the PTs had actually been injected into the arms of Canadians [6]. Due to the storage and logistical challenges of administering the Pfizer-BioNTech vaccine because of the extremely low storage temperature, the federal government has only distributed the Moderna vaccine to the three territorial governments responsible for residents in Canada's far north.

Canada has far less experience than Israel with emergency preparedness and response. As a consequence, the muscle memory exhibited by professionals, government public servants and the general public in Israel when engaging in emergency efforts is almost non-existent in Canada on any regional or national basis. Again, due to Canada’s decentralized federation, most of the emergency planning has been done at the PT level of government. While many PT governments have done creditable jobs, they have limited incentive to collaborate on pan-Canadian emergency planning, and no formalized intergovernmental agency responsible for national emergency planning. In fact, there are significant legislative and procedural limitations on the federal government’s use of its Emergencies Act and, thus far, the federal government has not declared a state of national state of emergency in the face of the pandemic [7].

There are also important health system differences. Unlike Israel, Canada has a fragmented and under-developed electronic medical record (EMR) system. This prevents national (and even individual PT) tracking to allow for effective identification and contact of priority individuals for vaccination with follow-up tracking afterwards. However, this challenge goes well beyond EMR infrastructure. Public health surveillance data are collected and used by the 13 PT governments using differing protocols and standards. These data are then stored in 13 individual PT databases defying efforts to track vaccination progress on a more national basis and to conduct more probative statistical analyses based on consistently defined data [8].

Another health system difference is Israel’s use of its well-developed primary care system to deliver vaccines. Except in Canada’s far north where publicly-managed primary health centres are the norm, the administration of vaccines has overly relied on hospitals because the majority of primary care physicians are independent contractors paid on a fee-for-service basis raising issues of coordination and cost. Canada has ended up relying on a more hospital-centric administration of vaccines for the general population due to the fragmented nature of primary care. To vaccinate residents living in long-term care (LTC) homes, Israel had relied on a single organization – Magen David Adom (MDA) – the country’s national medical emergency services organization [1]. Nothing similar exists in Canada. Instead, each PT has assembled its own approach and its own task teams to go into LTC facilities and administer vaccinations in the process sacrificing some speed in the process.

## Transferable policy lessons from Israel to Canada

Given the enormous differences between the two countries and their respective health systems, are there any transferable policy lessons from Israel to Canada? There are at least two obvious lessons.

The first is that the pandemic poses a clear and present danger to the security of all countries that in turn puts a premium on more unified and strategic planning and execution of vaccination strategies. Admittedly, this is easier to achieve in a unitary state like Israel, but with some effort should also be achievable in a more decentralized polity. It simply requires more innovative federal leadership than has been demonstrate so far in the co-crafting and co-implementation of a federal-provincial-territorial vaccination campaign. As leverage, the federal government should use its willingness to continue to pay for the country’s entire vaccine supply during the crisis. This leadership would include spearheading and rapidly implementing intergovernmental protocols to share vaccination and post-vaccination surveillance data [8]. While this does not require the use of federal emergency powers, it would nonetheless require the kind of focus among political leaders and public servants that the Government of Israel has exhibited throughout the pandemic.

Canada can also learn from the effectiveness of Israel’s primary care practitioners in delivering vaccine doses to the general population. Currently, Canada’s approach, where limited to hospitals, is too constricted and should be opened up so that primary care providers can play a more central role in the delivery of the vaccine. Although most providers are independent contractors and therefore not subject to government fiat, incentives can still be offered by PT governments so that these providers become key players in the vaccination campaign. As for long-term care facilities, if PT vaccination efforts are not successful by mid-February, the Government of Canada should offer the resources of the Canadian Armed Forces and their medical personnel to provide rapid vaccination services, similar to the emergency response it provided in addressing disastrous levels of COVID-19 infection and death in worst-hit LTC facilities in Ontario and Quebec in the first phase of the pandemic.

## Conclusions

Despite some profound political, geographic, demographic and health system differences with Israel, Canada can draw some important policy lessons to accelerate and improve its ongoing COVID-19 vaccination strategy. The first policy lesson is to emulate a more coherently national approach similar to Israel’s highly disciplined and focused approach but to do so through intergovernmental agreement. Such agreement, to be achieved quickly, would have to be aggressively levered through the federal government’s willingness to continue to underwrite the full cost of the vaccine and its storage until distributed to the provinces and territories. The second policy lesson is for Canada, like Israel, to use more of its primary care resources in administering COVID-19 vaccines to the general population and to rely on a single agency to vaccinate residents and workers in LTC facilities in situations where provincial and territorial efforts are not rapid enough.

## Data Availability

Not applicable.
