# Publisher Correction to: The rollout of the COVID-19 vaccination: what can Canada learn from Israel?

**DOI:** 10.1186/s13584-021-00456-y

**Published:** 2021-02-26

**Authors:** Gregory P. Marchildon

**Affiliations:** Institute of Health Policy, Management and Evaluation, University of Toronto, 155 College Street, Suite 425, Toronto, Ontario M5T 3M6 Canada

**Correction to: Isr J Health Policy Res 10, 12 (2021)**

**https://doi.org/10.1186/s13584-021-00449-x**

Following publication of the original article [1], the author identified an error which was introduced during the typesetting process. Table 1 was updated to show the updated vaccination doses administered. The values within the table were updated but the table description was not updated.

Below the incorrect and correct table description:

**Incorrect:** Table 1 Cumulative COVID-19 vaccination doses administered per 100 people by **Feb. 1**, 2021, or latest date available

**Correct:** Table 1: Cumulative COVID-19 vaccination doses administered per 100 people by **Feb. 11**, 2021, or latest date available

The original article has been updated.
